# Enhanced control of pathogenic *Vibrio* spp. in aquaculture using phages capable of disrupting biofilms outside their host range

**DOI:** 10.1128/aem.01673-25

**Published:** 2025-10-22

**Authors:** Ni Wang, Chengcheng Li, Jiulong Zhao, Yufei Yue, Tongmei Shi, Zengmeng Wang, Yantao Liang, Yongyu Zhang, Min Wang

**Affiliations:** 1 College of Marine Life Sciences, MoE Laboratory of Evolution and Marine Biodiversity, Institute of Evolution and Marine Biodiversity, Frontiers Science Center for Deep Ocean Multispheres and Earth System, Center for Ocean Carbon Neutrality, Ocean University of China 535359 , Qingdao, China; 2 Qingdao New Energy Shandong Laboratory, Key Laboratory of Biofuels, Shandong Provincial Key Laboratory of Energy Genetics, Qingdao Institute of Bioenergy and Bioprocess Technology, Chinese Academy of Sciences 85437 , Qingdao, China; 3 University of Chinese Academy of Sciences 590852 , Beijing, China; 4 Southern Marine Science and Engineering Guangdong Laboratory (Zhuhai), Zhuhai, China; 5 Haide College, Ocean University of China 12591, Qingdao, China; Indiana University Bloomington, Bloomington, Indiana, USA

**Keywords:** biofilm control, aquaculture, *Vibrio* spp, lytic phages, phage synergy

## Abstract

*Vibrio* species are among the most devastating pathogens in aquaculture, causing substantial economic losses. Their capacity to form resilient, often multispecies biofilms confers exceptional resistance to conventional antimicrobials. While bacteriophages represent a promising alternative for combating biofilm-associated infections, phages exhibiting potent antibiofilm activity against *Vibrio* spp. remain scarce. In this study, we isolated and characterized two novel lytic phages, VpT and VpR, specific to multidrug-resistant *Vibrio parahaemolyticus*. Genomic and phylogenetic analyses identified VpT and VpR as members of a novel viral family and a novel species within the class *Caudoviricetes*, respectively. Remarkably, beyond exhibiting potent biofilm prevention and disruption capabilities against their host strains, both phages effectively degraded preformed biofilms by eight non-host *Vibrio* species as well as complex multi-species *Vibrio* communities. This host range-independent, cross-species biofilm degradation activity highlights their potential for controlling complex polymicrobial biofilms. Moreover, VpT and VpR appear to employ distinct infection strategies, and when applied as a cocktail, they synergistically enhanced antimicrobial efficacy by suppressing bacterial regrowth, thereby addressing a key limitation of phage therapy. Our findings establish these phages as promising biocontrol agents against *Vibrio* infections and biofilm-associated challenges in aquaculture systems.

**IMPORTANCE:**

The effective control of *Vibrio* infections in aquaculture remains a major challenge, largely due to the resilience of multispecies biofilms against conventional antimicrobial treatments. This study identifies two novel lytic phages, VpT and VpR, which not only prevent and disrupt biofilms formed by phage-sensitive, multidrug-resistant *Vibrio parahaemolyticus* but also degrade preformed biofilms of multiple phage-insensitive *Vibrio* species and complex mixed-species *Vibrio* communities. Notably, their distinct and synergistic infection mechanisms enhance antimicrobial efficacy by suppressing bacterial regrowth, thereby addressing a critical limitation of current phage therapies. These findings underscore the strong potential of these phages as next-generation biocontrol agents for combating complex *Vibrio* biofilms and enhancing pathogen control in aquaculture systems.

## INTRODUCTION

*Vibrio* species rank among the most devastating pathogens in aquaculture, causing substantial economic losses worldwide (1, 2). A major contributor to their virulence is their ability to form biofilms, structured microbial communities encased in a self-produced extracellular matrix (3, 4). These biofilms enhance bacterial adhesion to host surfaces and increase infection efficiency, as demonstrated in *Vibrio alginolyticus* (5), *Vibrio harveyi* (6, 7), *Vibrio parahaemolyticus* (8, 9), and *Vibrio vulnificus* (10). They also persist on a variety of surfaces including seafood products such as crabs, oysters, and shrimp, as well as on abiotic surfaces in aquaculture systems (11–13). Acting as robust protective barriers, these biofilms impede antimicrobial penetration and enable bacterial evasion of host immune responses (14–17), allowing bacterial populations to survive antibiotic concentrations up to 1,000-fold higher than those effective against planktonic cells (18, 19). This heightened resistance makes biofilms a major factor in persistent and recurrent infections that are notoriously difficult to eradicate (3, 20).

Bacteriophages (phages) have emerged as promising alternatives to antibiotics, particularly for combating antibiotic-resistant bacteria (21, 22) and biofilm-associated infections (23–26). Their antibiofilm efficacy is mediated through multiple mechanisms (27, 28): (i) induction of host-derived extracellular polymeric substances (EPS)-degrading enzymes during infection (29); (ii) expression of phage-encoded depolymerases that break down EPS components such as polysaccharides, lipids, and proteins (30); and (iii) migration through aqueous channels within the biofilm to access and lyse embedded bacterial cells (31). By disrupting the biofilm matrix, phages can also enhance the penetration and efficacy of conventional antimicrobials, offering a synergistic approach to biofilm eradication (32–34).

Despite growing interest in phage-based biofilm control, phages with robust biofilm-disrupting activity against *Vibrio* species remain poorly characterized (35). This challenge is further complicated by the aquaculture-associated systems, which often involve multispecies bacterial communities that confer increased resilience and resistance to treatment (36–38). In this study, we isolated and characterized two novel lytic phages, VpT and VpR, targeting a multidrug-resistant *V. parahaemolyticus* strain, a pathogen of critical concern in both aquaculture and food safety (39–42). Both phages displayed potent anti-biofilm activity, acting not only on their host strains but also on multiple phage-insensitive *Vibrio* species and mixed-species communities. Remarkably, the VpT/VpR cocktail synergistically suppressed bacterial regrowth, overcoming a key limitation of phage therapy. These findings highlight VpT and VpR as promising candidates for phage-based biocontrol of biofilm-associated *Vibrio* infections in aquaculture.

## RESULTS

### Characteristics of phages VpT and VpR lytic to multidrug-resistant *V. parahaemolyticus*

#### Morphology characterization of phages VpT and VpR

**Fig 1:**
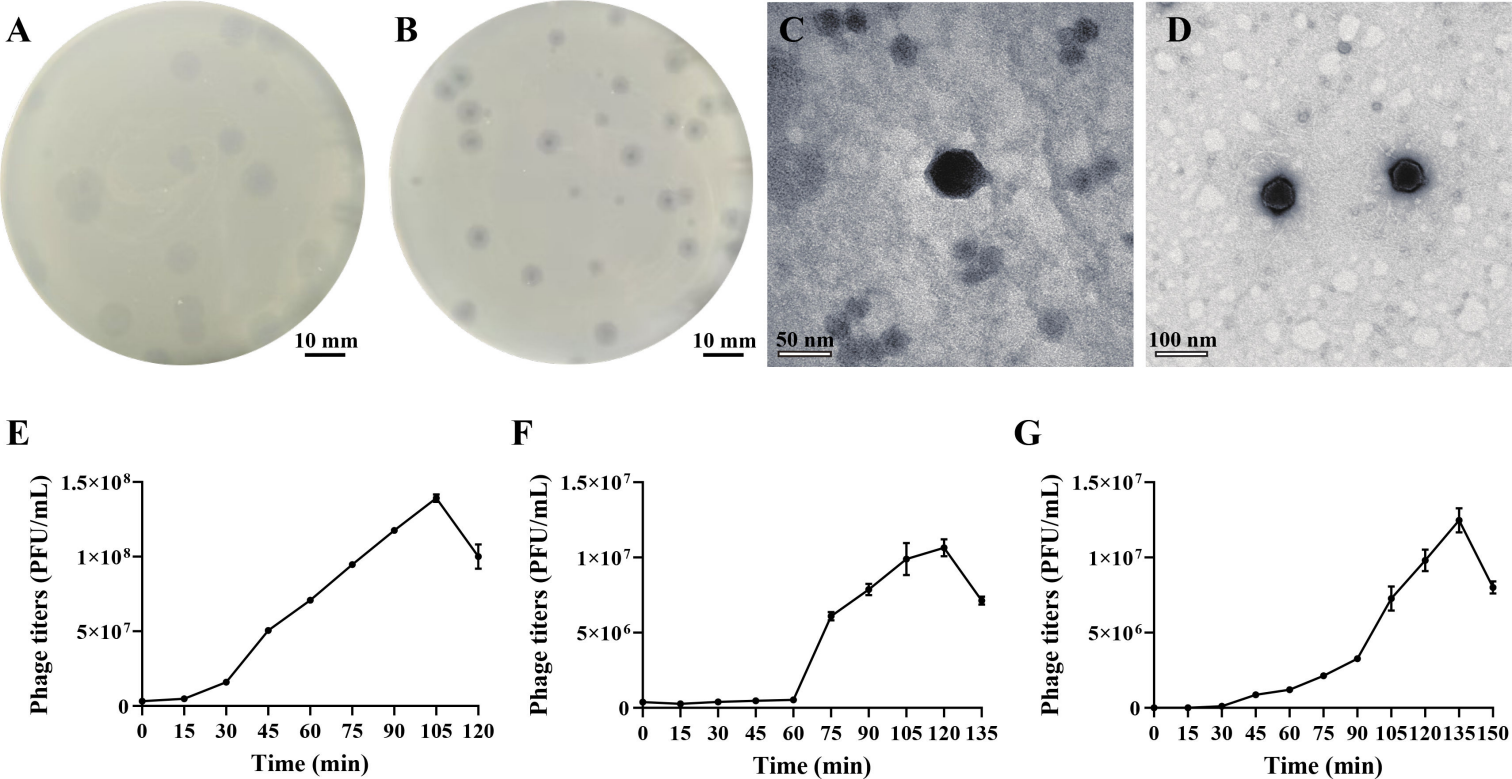
Morphology and infection dynamics of *Vibrio* phages VpT and VpR. Plaque morphology of phages VpT (**A**) and VpR (**B**) on *V. parahaemolyticus* 108T lawns, TEM micrographs of VpT (**C**) and VpR (**D**), one-step growth curves of VpT (**E**) and VpR (**F**) during infection of *V. parahaemolyticus* 108T, one-step growth curve of VpR (**G**) during infection of *V. parahaemolyticus* 108R.

*V. parahaemolyticus* 108T was characterized as a multidrug-resistant pathogen, exhibiting resistance to multiple antibiotics, including ampicillin, amikacin, carbenicillin, tetracycline, minocycline, clindamycin, penicillin, oxacillin, piperacillin, and vancomycin (Table S1). Phages VpT and VpR were subsequently isolated using *V. parahaemolyticus* 108T and its derivative strain 108R (resistant to VpT infection) as respective hosts. On double-layer agar plates, VpT and VpR produced distinct, clear circular plaques with diameters of 7.4 ± 0.8 mm and 5.0 ± 0.8 mm, respectively, after 12 hours of incubation (Fig. 1A and B). Notably, VpR plaques exhibited a clear central lysis zone surrounded by fuzzy halos (Fig. 1B), suggesting the potential presence of depolymerase activity (43).

Transmission electron microscopy (TEM) analysis revealed that phage VpT possessed an icosahedral head with a diameter of 50 ± 5 nm and a short tail measuring 8 ± 4 nm in length (Fig. 1C). Similarly, phage VpR featured an icosahedral head with a diameter of 28 ± 3 nm and a short tail measuring 6 ± 3 nm in length (Fig. 1D). Based on their morphological characteristics, both phages belonged to podovirus.

#### Infection characterization of phages VpT and VpR

One-step growth curve analyses revealed distinct life cycle parameters of phages VpT and VpR. When infecting *V. parahaemolyticus* 108T, VpT exhibited rapid infection kinetics, characterized by a short latent period of 15 minutes, a burst size of 44 PFU/cell, and a complete lytic cycle of 105 minutes (Fig. 1E). In contrast, VpR demonstrated a longer latent period of 60 minutes and a substantially higher burst size of 285 PFU/cell with a total lytic cycle time of 120 minutes (Fig. 1F). Notably, the VpT-resistant strain 108R remained susceptible to VpR, which completed a lytic cycle with a 30-minute latent period and a burst size of 125 PFU/cell (Fig. 1G), indicating divergent infection strategies between these two phages.

The host range of each phage was further evaluated against a panel of 30 *Vibrio* strains (Table 1). Phage VpT exhibited a narrow host range, showing lytic activity only against *V. parahaemolyticus* 108T and *V. parahaemolyticus* VIB611, which are distinct strains differing in biofilm-forming capacity. In contrast, VpR displayed a slightly broader host range, lysing *V. parahaemolyticus* 108T, VIB611, and the phage-resistant mutant strain 108R. Additionally, chloroform treatment had no impact on the infectivity of either phage, indicating the absence of lipid envelope components in their virion structures (44) (Fig. S1).

**TABLE 1:** Lytic and antibiofilm efficacy of individual phages (VpT and VpR) and their combination against 30 *Vibrio* spp. isolates^*a*^

Strain	Most closely related strain	Accession	Similarity	Lytic activity of VpT	Lytic activity of VpR	Lytic activity of VpT/VpR	Biofilm-forming capacity of bacterial strains	Antibiofilm efficacy of individual phages and their combination	
								Biofilm prevention	Biofilm degradation
108T	*V. parahaemolyticus*	ATCC 17802	99.85%	+	+	+	+	+	+
108R	*V. parahaemolyticus*	ATCC 17802	99.85%	−	+	+	+	− (VpT); + (VpR); + (VpT/VpR)	− (VpT); + (VpR); + (VpT/VpR)
VIB611	*V. parahaemolyticus*	ATCC 17802	99.51%	+	+	+	+	+	+
16872	*V. parahaemolyticus*	ATCC 17802	99.30%	−	−	−	+	−	+
TFB102	*V. parahaemolyticus*	NBRC 12711	99.85%	−	−	−	−	/	/
TFB109	*V. parahaemolyticus*	NBRC 12711	99.56%	−	−	−	−	/	/
XFB1002	*V. parahaemolyticus*	NBRC 12711	99.51%	−	−	−	+	−	−
FB1015	*V. parahaemolyticus*	NBRC 12711	99.24%	−	−	−	+	−	+
FB1016	*V. parahaemolyticus*	NBRC 12711	99.78%	−	−	−	+	−	−
FB1017	*V. parahaemolyticus*	NBRC 12711	99.10%	−	−	−	+	−	−
17X-5-1	*V. harveyi*	NBRC 15634	99.72%	−	−	−	+	−	−
VIB-645	*V. harveyi*	NBRC 15634	100.00%	−	−	−	+	−	−
VIB-283	*V. alginolyticus*	ATCC 17749	100.00%	−	−	−	+	−	+
05-1	*V. alginolyticus*	ATCC 17749	99.58%	−	−	−	+	−	−
VIB462	*V. alginolyticus*	ATCC 17749	99.31%	−	−	−	+	−	−
1807	*V. alginolyticus*	ATCC 17749	99.30%	−	−	−	+	−	−
18-1	*V. campbellii*	CAIM 519	100.00%	−	−	−	+	−	+
VIB463	*V. campbellii*	CAIM 519	99.36%	−	−	−	+	−	−
VIB797	*V. campbellii*	CAIM 519	99.22%	−	−	−	+	−	+
0058	*Vibrio sagamiensis*	LC2-047	99.15%	−	−	−	+	−	−
VIB612	*Vibrio sagamiensis*	LC2-047	99.46%	−	−	−	+	−	−
FB1006	*V. hyugaensis*	090810a	99.49%	−	−	−	+	−	−
HNX006	*V. hyugaensis*	090810a	99.57%	−	−	−	+	−	−
yc6	*V. hyugaensis*	090810a	99.27%	−	−	−	+	−	−
HNXS004	*V. hyugaensis*	090810a	99.06%	−	−	−	+	−	+
FB1008	*V. hyugaensis*	090810a	99.49%	−	−	−	+	−	+
16655	*V. neocaledonicus*	NC470	99.79%	−	−	−	+	−	−
16578	*V. neocaledonicus*	NC470	99.22%	−	−	−	+	−	−
16842	*V. neocaledonicus*	NC470	99.65%	−	−	−	+	−	+
X10S	*V. neocaledonicus*	NC470	99.93%	*−*	−	−	+	−	−

^*a*^ +, active; −, inactive; /, not determined.

### Phages VpT and VpR effectively inhibit biofilm formation in their host strains

**Fig 2:**
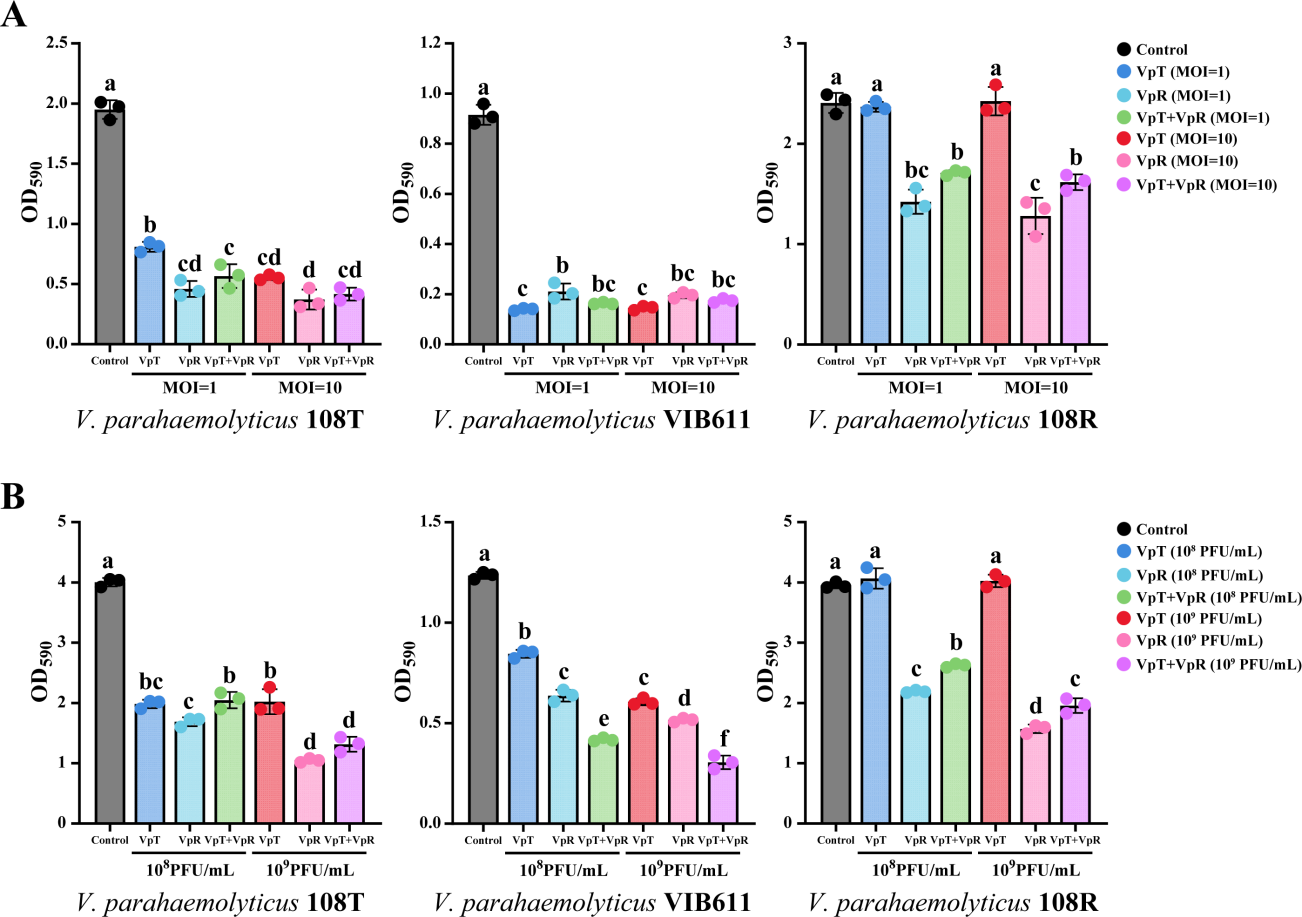
Antibiofilm activities of phages VpT, VpR, and VpT/VpR cocktail against *V. parahaemolyticus* strains 108T, VIB611, and 108R. (**A**) Inhibition of biofilm formation by VpT, VpR, and VpT/VpR cocktail at MOIs of 1 and 10; (**B**) disruption of pre-formed mature biofilms by VpT, VpR, and VpT/VpR cocktail at final concentrations of 10^8^ and 10^9^ PFU/mL. Different letters represent significant statistical differences (*P* < 0.05) between groups.

The biofilm-inhibitory effects of phages VpT, VpR, and their combination were evaluated against their host strains (*V. parahaemolyticus* 108T, *V. parahaemolyticus* VIB611, and *V. parahaemolyticus* 108R), as well as phage-insensitive strains with strong biofilm-forming capacity (Table 1). Crystal violet quantification demonstrated comparable inhibitory effects at multiplicity of infection (MOI) = 1 and MOI = 10 in the susceptible strains, with OD_590_ values decreasing from 2.0 to 0.6 for *V. parahaemolyticus* 108T (a 68.7% reduction) and from 0.9 to 0.2 for *V. parahaemolyticus* VIB611 (an 81.5% reduction) (Fig. 2A, *P* < 0.05). Notably, only VpR alone and the phage combination significantly inhibited biofilm formation in *V. parahaemolyticus* 108R, reducing OD_590_ from 2.4 to 1.5 (a 38.1% reduction) (Fig. 2A, *P* < 0.05). In contrast, neither phage produced a significant inhibitory effect on biofilm formation in non-susceptible strains (Table 1; Fig. S2), confirming the host-specific nature of their inhibitory activity.

### Phages VpT and VpR disrupt mature biofilms across diverse *Vibrio* species

The biofilm-disrupting potential of phages VpT, VpR, and their combination was further evaluated against mature biofilms formed by diverse *Vibrio* strains (Table 1). All phage treatments significantly disrupted established biofilms of *V. parahaemolyticus* 108T and *V. parahaemolyticus* VIB611, achieving 54 ± 16% reduction in OD_590_ compared to untreated controls (Fig. 2B, *P* < 0.05). In contrast, only VpR and the VpT + VpR cocktail effectively disrupted the mature biofilm of strain 108R, yielding a 47 ± 11% reduction in OD_590_ compared with controls (Fig. 2B, *P* < 0.05). Moreover, disruption was significantly greater at a phage concentration of 10^9^ PFU/mL than at 10^8^ PFU/mL in the susceptible strains (Fig. 2B), except for VpT against strains 108T and 108R, indicating that higher phage concentration is generally more effective for biofilm disruption.

**Fig 3:**
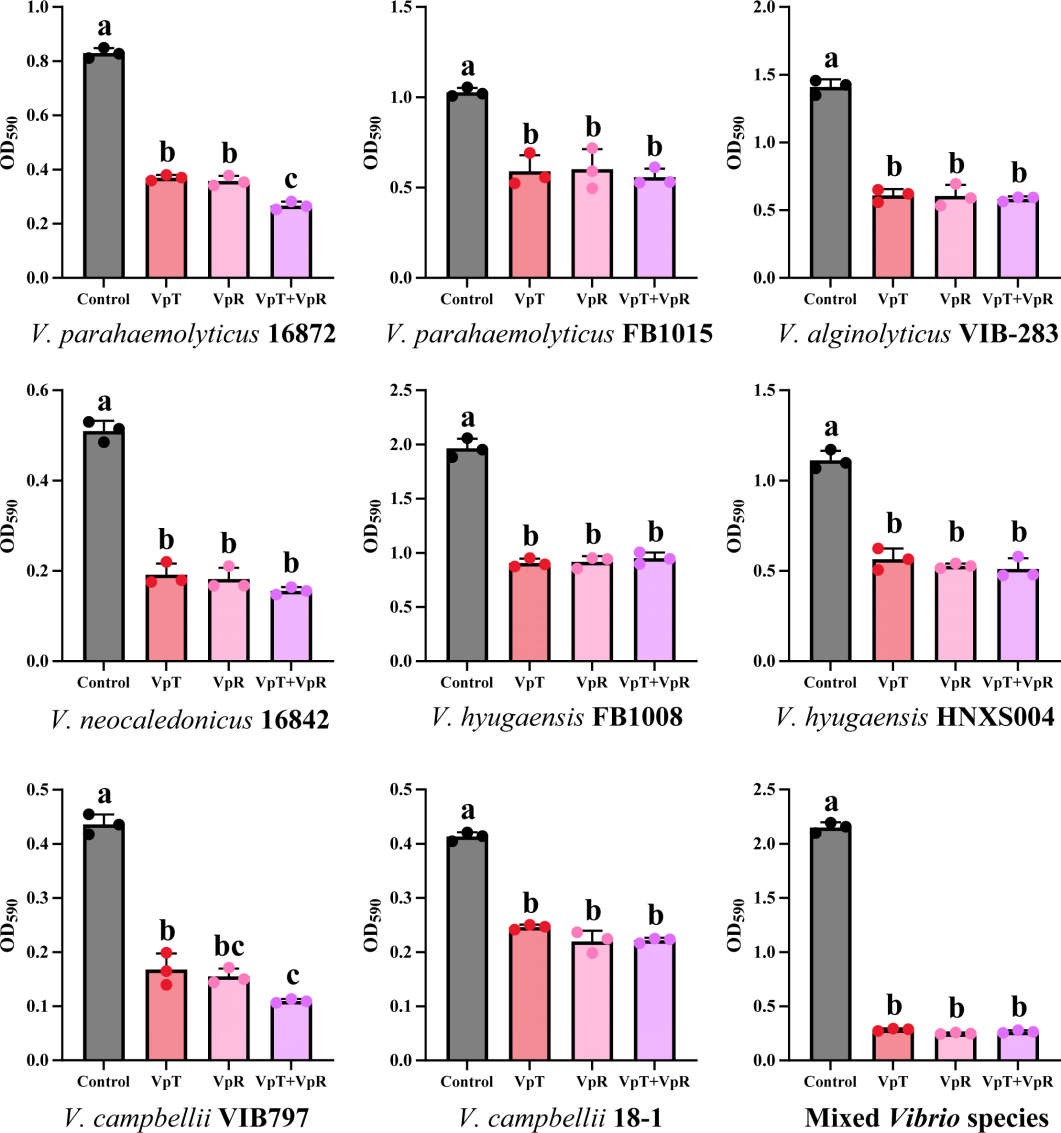
Biofilm degradation activity of phages VpT, VpR, and VpT/VpR cocktail (10^9^ PFU/mL) against multiple non-host *Vibrio* species and mixed-species *Vibrio* communities. Different letters represent significant statistical differences (*P* < 0.05) between groups.

Notably, substantial biofilm degradation was also observed against eight phage-insensitive strains, including *V. alginolyticus* VIB-283, *Vibrio campbellii* 18-1, *V. campbellii* VIB797, *Vibrio hyugaensis* FB1008, *V. hyugaensis* HNXS004, *Vibrio neocaledonicus* 16842, as well as *V. parahaemolyticus* 16872 and *V. parahaemolyticus* FB1015. In these cases, treatments with VpT, VpR, or their cocktail led to 40%–75% reductions in OD_590_ values (Fig. 3, *P* < 0.05). These findings demonstrate that the biofilm-degrading activity of VpT and VpR operates independently of their lytic host range, revealing a broad-spectrum, cross-species antibiofilm potential. Furthermore, while crystal violet quantification showed that neither individual phages nor their cocktail significantly inhibited the formation of mixed-species biofilms (Fig. S2), all treatments effectively disrupted pre-established biofilms, achieving a substantial reduction (88 ± 1%) in OD_590_ compared to the untreated control (Fig. 3; *P* < 0.05). This robust efficacy against mature, mixed-species biofilm communities underscores the potential applicability of these phages in complex aquaculture environments.

### Synergistic antibacterial activity of phages VpT and VpR provides immediate and sustained suppression of *V. parahaemolyticus* growth

**Fig 4:**
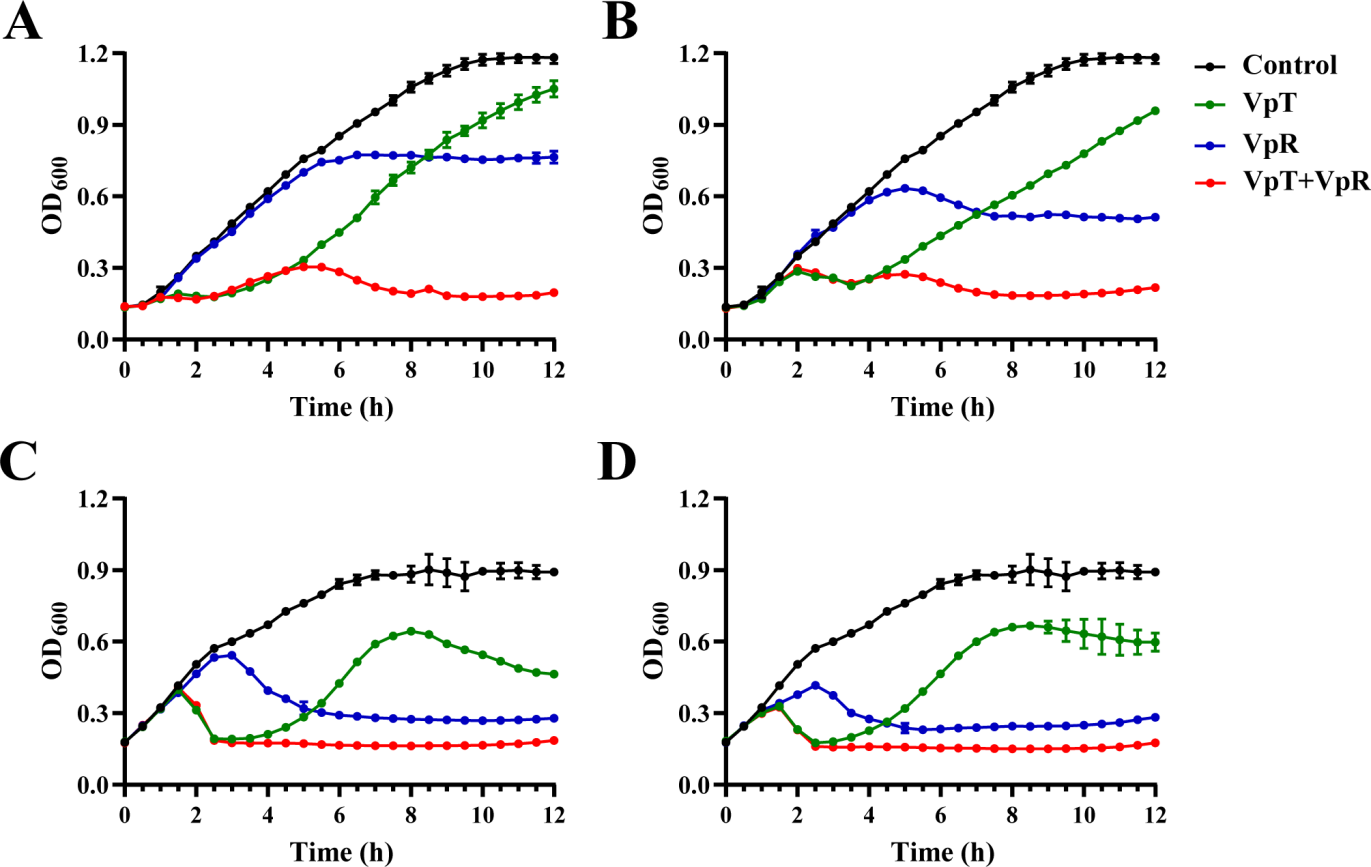
Growth curves of *V. parahaemolyticus* 108T treated with phages VpT, VpR, and VpT/VpR cocktail at MOIs of 0.01 (**A**), 0.1 (**B**), 1 (**C**), and 10 (**D**).

The antibacterial efficacy of phages VpT and VpR, individually and in combination, was evaluated by monitoring the growth dynamics of *V. parahaemolyticus* 108T at MOIs of 0.01, 0.1, 1, and 10 (Fig. 4). In untreated control cultures, bacteria exhibited robust growth, with increasing OD_600_ values that eventually plateaued. Treatments with VpT alone resulted in rapid but transient lysis, with OD_600_ decreasing sharply after 1.5–2 hours at all MOIs. However, bacterial regrowth was observed shortly thereafter, with increasing OD_600_ values, likely due to the emergence of phage-resistant variants (45). In contrast, VpR exhibited delayed yet sustained suppression, initiating OD_600_ reduction at 7, 5, 3, and 2.5 hours post-infection with increasing MOI and effectively inhibiting bacterial growth throughout the 12-hour period. At 12 hours, bactericidal activity was significantly higher at MOIs of 1–10 (40.6% for VpT, 68.6% for VpR) compared to MOI of 0.1 (18.9% for VpT, 56.7% for VpR) and MOI of 0.01 (11.1% for VpT, 35.3% for VpR).

Notably, the VpT and VpR combination synergized the strengths of both phages. The phage cocktail induced rapid lysis (~2 hours) similar to VpT alone, while simultaneously preventing the emergence of resistant populations, as achieved by VpR. This combination consistently maintained superior bacterial suppression, with OD_600_ values remaining below 0.3 at all MOIs throughout the 12-hour experiment, significantly outperforming either phage used alone. Moreover, bacterial suppression was more stable at MOIs of 1–10, whereas a minor rebound in OD_600_ was observed at ~5 hours under lower MOIs (0.01 and 0.1), further supporting MOI = 1 as the minimal effective concentration for phage combination therapy.

### Genomic and phylogenetic analysis of VpT and VpR

**Fig 5:**
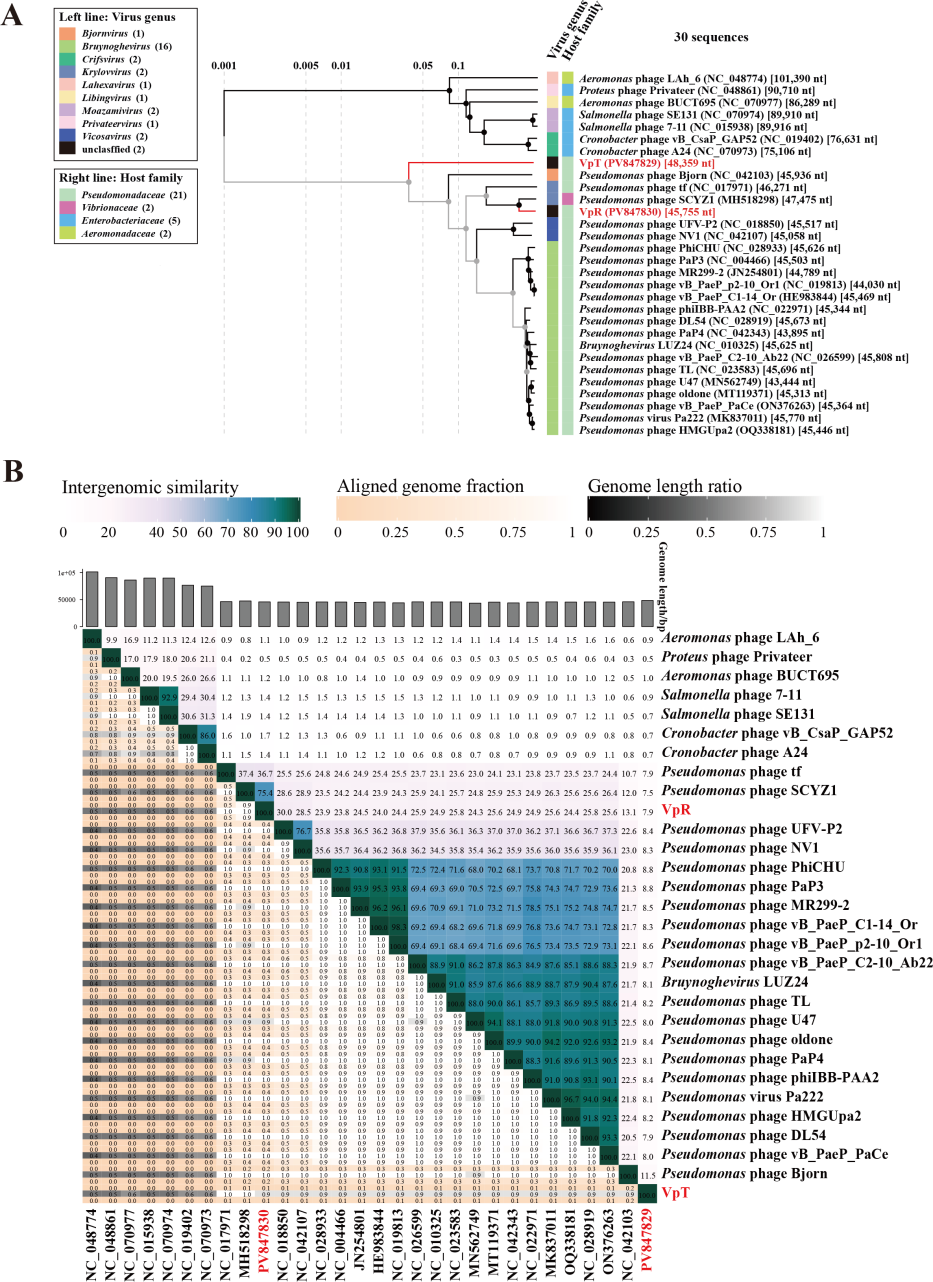
Bioinformatics characteristics of phages VpT and VpR. (**A**) Phylogenetic tree including VpT, VpR, and other phages generated using ViPTree; (**B**) heatmap showing ANI values between VpT, VpR, and related phages, calculated using VIRIDIC.

Whole-genome sequencing revealed that phages VpT and VpR possess circular dsDNA genomes of 48,359 and 45,755 bp, with G+C contents of 47.99% and 52.25%, respectively (Table S2). BLASTn-based nucleotide sequence comparison demonstrated limited genomic similarity between VpT and previously characterized phages, with the closest match being *Pseudomonas* virus Pa222 (73.01% identity, 0% coverage). VIRIDIC analysis further confirmed VpT’s genomic novelty, showing average nucleotide identity (ANI) of only 0.5%–11.5% with 29 related phages. In contrast, VpR exhibited strong homology to *Pseudomonas* phage SCYZ1, sharing 87.07% nucleotide identity across 87% query coverage, with an ANI of 75.4% as determined by VIRIDIC. Phylogenetic and comparative genomic analysis (Fig. 5A and B) classified VpT as a member of a novel viral family within *Caudoviricetes*, whereas VpR was identified as a novel species within the same genus as *Pseudomonas phage* SCYZ1.

**Fig 6:**
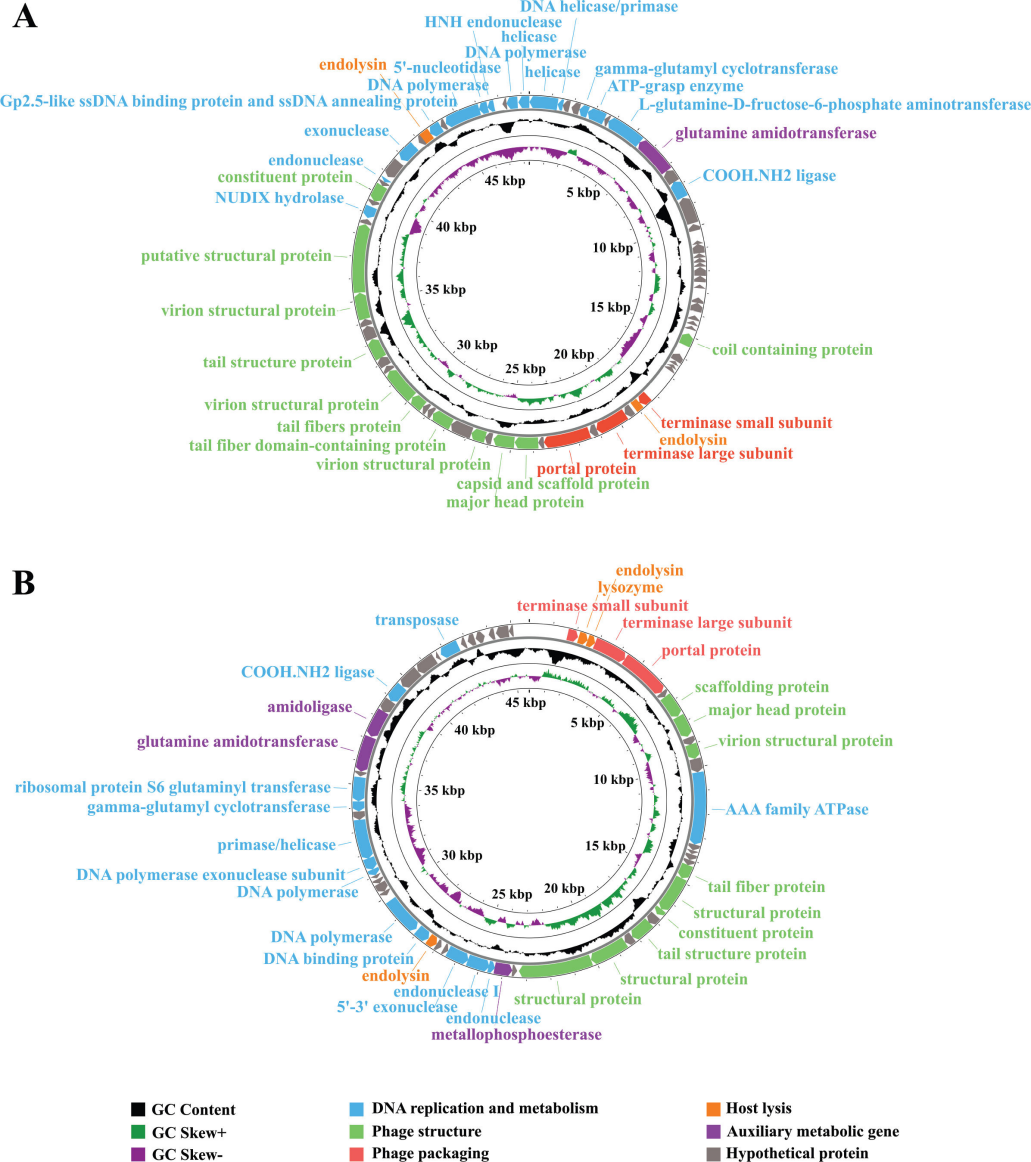
Circular genome maps and protein functional annotation of phages VpT (**A**) and VpR (**B**). Arrows indicate the positions and strand orientation of individual ORFs.

Genomic analysis predicted 73 and 60 open reading frames (ORFs) in phages VpT and VpR, respectively, with 43.8% (VpT) and 51.7% (VpR) assigned putative functions (Table S2). These ORFs were organized into five conserved functional modules: (i) DNA replication and regulation (ii), structural components (iii), packaging machinery (iv), host lysis systems (v), and auxiliary metabolic genes (Fig. 6A and B; Tables S3 and S4). Notably, VpR encodes a metallophosphoesterase (ORF26), which may counteract host bacterial antiviral immunity (46) and mediate the dephosphorylation of certain proteins to allow more effective production of phage (47). Moreover, both phages encode predicted biofilm-controlling proteins, including tail fiber proteins (VpT, ORF44/47; VpR, ORF17), lysozyme (VpR, ORF2), and endolysins (VpT, ORF33/65; VpR, ORF3/32), which likely contribute to their observed antibiofilm capabilities (31).

Additionally, each phage encodes a single tRNA gene, with biosafety analyses confirming the absence of virulence factors, antibiotic resistance genes, integrases, and lysogeny-associated elements, supporting their potential as safe biocontrol agents against *Vibrio* infections.

## DISCUSSION

*Vibrio* species are among the most significant pathogenic threats in aquaculture systems, with many strains capable of forming protective biofilms that exhibit high resistance to conventional antibacterial treatments (6, 48). These biofilms contribute to persistent infections and substantially complicate disease management (49–51). While bacteriophages have emerged as promising biofilm control agents in various contexts (52–54), their specific application against *Vibrio* biofilms remains poorly explored.

In this study, we isolated and characterized two lytic phages, VpT and VpR, targeting a multidrug-resistant *V. parahaemolyticus* strain. Both phages exhibited potent anti-biofilm activity, not only against their hosts but also across multiple phage-insensitive *Vibrio* species and mixed-species *Vibrio* communities. This cross-species biofilm degradation activity is particularly valuable in aquaculture settings, where aquatic hosts are frequently co-infected with complex *Vibrio* consortia that form resilient, multispecies biofilms (55–60). For instance, *V. harveyi*, *V. parahaemolyticus*, and *Vibrio fluvialis* have been simultaneously isolated from *Penaeus monodon* exhibiting red disease syndrome (55), while up to nine *Vibrio* species including *V. harveyi*, *V. vulnificus*, and *V. alginolyticus* have been reported in co-infected marine fish (58). In this context, phages such as VpT and VpR, which exhibit dual functionality by preventing biofilm formation and degrading established multispecies biofilms, represent a highly promising strategy for *Vibrio* control in aquaculture.

Phage-encoded lytic enzymes, such as polysaccharide depolymerases, endolysins, and virion-associated peptidoglycan hydrolases (61, 62), can degrade bacterial surface polymers including capsular polysaccharides, lipopolysaccharides, and peptidoglycan (63). They can also hydrolyze the EPS matrix of biofilms (64), thereby rapidly compromising essential structural components and disrupting biofilm integrity (65). Genomic analysis predicted that phages VpT and VpR encode such proteins, including tail fiber proteins with putative depolymerase activity, lysozyme, and endolysins, consistent with the halo zones observed in VpR plaques, which likely contribute to their biofilm inhibition and disruption activity. In phage-insensitive strains, these enzymes may act primarily on the extracellular matrix between bacterial cells, promoting biofilm degradation. Supporting this, endolysins from *Acinetobacter* phage AM24, *Acinetobacter* phage AP22, *Escherichia* phage ECD7, and *Enterobacteria* phage UAB_Phi87 can act beyond phage host specificity and non-specifically disrupt the peptidoglycan, extracellular DNA, and exopolysaccharides in biofilm formed by taxonomically distinct genera such as *Acinetobacter baumannii* and *Klebsiella pneumoniae* (66), irrespective of bacterial susceptibility to the corresponding phages. These findings highlight the potential of phage-derived enzymes for degrading established, multispecies biofilms when applied exogenously. Nevertheless, during biofilm development, intact phages may fail to bind their specific surface receptors and instead become entrapped within the dense bacterial community and extracellular matrix, which imposes steric barriers that restrict access to target substrates and allow biofilm formation to proceed unchecked (67–69).

A major limitation of phage therapy is the rapid emergence of phage-resistant bacterial variants. Combining phages with complementary infection mechanisms has proven effective in mitigating resistance (70, 71). Traditional methods for constructing phage cocktails, such as random screening or phage adaptation, are often labor-intensive and time-consuming (72, 73). Here, we applied a targeted approach (74) by isolating VpR using a phage-resistant derivative of *V. parahaemolyticus* 108T (designated 108R) that had evolved under selective pressure from VpT infection. VpT and VpR appear to employ distinct infection strategies, as supported by adsorption assays showing that VpR binds to both wild-type *V. parahaemolyticus* 108T and its phage-resistant variant, whereas VpT exhibits specificity only for the wild-type host (Fig. S3). This differential receptor usage likely contributes to the efficacy of the VpT/VpR cocktail in suppressing bacterial regrowth, which is a critical limitation of conventional phage therapy.

Beyond whole-phage applications, phage-derived antibacterial enzymes offer distinct advantages, including targeted effects and a reduced risk of inducing bacterial resistance (75–77). These enzymes, particularly those with biofilm-degrading capabilities, can be used in combination with other antimicrobials (including phages) to disrupt the biofilm matrix, enhance antimicrobial penetration, and improve bacterial clearance (64, 78). The genomes of VpT and VpR encode putative biofilm-degrading enzymes that merit further validation and application-oriented investigation.

Collectively, these findings highlight the potential of phages with broad-spectrum antibiofilm activity, particularly those effective beyond their host range, and employing complementary infection strategies as powerful biocontrol agents for pathogen control in aquaculture.

## MATERIALS AND METHODS

### Antibiotic susceptibility analysis of *V. parahaemolyticus* 108T

The antibiotic susceptibility of *V. parahaemolyticus* 108T was assessed using the disk diffusion method (79) with commercially prepared antibiotic-impregnated disks (Hangzhou Microbial Reagent). Briefly, the bacterial strain was cultured in rich organic (RO) medium (1% tryptone, 1% yeast extract, 1% sodium acetate in artificial seawater, pH 7.8–8.0) at 28°C with shaking. A 100 µL of log-phase bacterial suspension (OD_600_ = 0.6) was evenly spread onto the surface of RO agar plates (1.5% agar). After complete absorption of the suspension, antibiotic disks were placed on the agar surface. The plates were then incubated at 30°C for 16–18 hours. Following incubation, the diameters of inhibition zones were measured to evaluate antibiotic sensitivity according to the Clinical and Laboratory Standards Institute (CLSI) guidelines (80, 81).

### Isolation and purification of phages

Phage VpT was isolated from coastal aquaculture seawater (Qingdao, China) using *V. parahaemolyticus* 108T as the host. The host strain was cultured in RO medium at 28°C with shaking. Upon reaching log-phase growth, water samples collected from a coastal aquaculture in Qingdao, China, were filtered through 0.22 µm sterile membranes and added to the bacterial cultures at a 10% (vol/vol) ratio. The mixtures were incubated at 28°C with shaking, and samples were collected and filtered daily for 7 consecutive days. Phage presence was confirmed using the double-agar overlay plaque assay.

Phage-resistant strains were generated by exposing *V. parahaemolyticus* 108T to phage VpT on double-layer agar plates until colonies emerged within plaques. Following four rounds of bacterial purification and spot test confirmation, the 16S rRNA gene of a representative isolate was amplified and sequenced, confirming its identity as a stable phage-resistant mutant, designated *V. parahaemolyticus* 108R. Phage VpR was isolated from coastal sediment in Qingdao, China, using *V. parahaemolyticus* 108R as the host, following the same enrichment and isolation procedures described above. All bacterial strains and purified phages were preserved in liquid medium supplemented with glycerol to a final concentration of 25% (vol/vol) at −80°C for long-term storage.

### Morphological characterization of phages

Phage morphology was examined using negative staining and TEM as previously described (82). Briefly, a 10 µL aliquot of the enriched phage suspension was adsorbed onto a 200-mesh copper grid for 1 minute. Excess liquid was removed, and the grid was stained with 9 µL of 2% uranyl acetate (pH 4.5) for 3 minutes. After air-drying, samples were visualized using a Hitachi H-7650 TEM operated at 80 kV. Images were acquired using the Gatan Inc. system, and phage morphological features, including head and tail dimensions, were analyzed using ImageJ v2.9.0.

### One-step growth curve determination

Phage growth kinetics were determined using a modified one-step growth curve assay as previously described (83). Briefly, 1 mL of log-phase host bacteria was mixed with phage lysate at a MOI of 0.01 and incubated at 28°C with shaking (160 rpm) for 20 minutes to allow adsorption. Unadsorbed phages were removed by centrifugation at 8,000 × *g* for 3 minutes at 4°C, followed by two successive washes with 1 mL RO liquid medium. The resulting pellet was resuspended in 1 mL of RO medium, and a 30 µL aliquot of the final suspension was inoculated into 30 mL of RO medium and incubated at 28°C with continuous shaking. Phage titers were determined at 15-minute intervals using the double-layer agar plaque assay. The burst size was calculated as the ratio of progeny phage particles released after the lytic cycle to the initial number of infecting phages.

### Host range determination and chloroform sensitivity testing

Phage host range was evaluated using spot assays (84) against 30 *Vibrio* strains (Table 1). Briefly, 1  mL of each log-phase bacterial culture was mixed with 5 mL of molten RO soft agar (0.5% agar, 48°C) and overlaid onto RO solid agar plates. Following solidification, 5 µL of phage lysate was spotted onto the surface. Plates were incubated overnight at 28°C and examined for clear lytic zones to determine phage infectivity against each *Vibrio* strain.

For chloroform sensitivity testing, 1 mL of filtered phage lysate was mixed with 0%, 1%, or 5% (vol/vol) chloroform and vortexed for 1 minute. The mixtures were then incubated at room temperature for 30 minutes. After centrifugation at 6,000 × *g* for 5 minutes, the supernatants were spotted onto *V. parahaemolyticus* 108T lawn plates. Plates were incubated overnight at 28°C, and plaque formation was assessed to determine phage sensitivity to chloroform.

### Quantitative assessment of phage-mediated biofilm inhibition and disruption

The biofilm-forming capacity of *Vibrio* strains was assessed using the crystal violet staining assay. Briefly, 10 µL of log-phase *Vibrio* cultures were inoculated into 190 µL of fresh RO medium in 96-well plates and incubated statically at 30°C for 24 hours. After incubation, planktonic cells were removed, and the wells were gently washed twice with sterile phosphate buffered saline to remove non-adherent cells. The remaining biofilms were stained with 200 µL of 0.1% (wt/vol) crystal violet for 10 minutes, rinsed with distilled water, and subsequently solubilized with 200 µL of 33% (vol/vol) glacial acetic acid. Biofilm biomass was quantified by measuring absorbance at 590 nm (OD_590_) using a microplate reader. For each strain, the mean OD of three replicates was used to determine biofilm-forming capacity. The cut-off value (ODc) was defined as the negative control mean plus three standard deviations, and strains were classified as non-biofilm (OD ≤ ODc), weak (ODc < OD ≤ 2 × ODc), moderate (2 × ODc < OD ≤ 4 × ODc), or strong producers (OD > 4 × ODc). Weak, moderate, and strong producers were considered biofilm-forming, whereas only non-biofilm producers were deemed incapable of biofilm formation (85, 86).

Phage-mediated biofilm inhibition and disruption were assessed against both monospecies and mixed-species biofilms (comprising 30 *Vibrio* strains listed in Table 1) following previously described methods with minor modifications (87, 88). For the biofilm inhibition assay, 10 µL of log-phase bacterial suspension (final concentration is approximately 10^6^ CFU/mL), and 10 µL of phage lysate (either single or combined) was added to 180 µL of RO medium in 96-well plates. Phage treatments were tested at MOIs of 1 and/or 10. Control wells received bacterial suspension without phages. Plates were incubated statically at 30°C for 24 hours, after which biofilm biomass was quantified using the crystal violet staining assay.

To evaluate phage-mediated disruption of mature biofilms, 10 µL of log-phase bacterial suspension (final concentration is approximately 10^6^ CFU/mL) was inoculated into 190 µL of RO medium in 96-well plates. Then, biofilms were grown statically for 24 hours at 30°C to establish mature biofilms. The established biofilms were then treated with either individual phages or a phage cocktail at final concentrations of 10^8^ PFU/mL and/or 10^9^ PFU/mL, followed by incubation at 30°C for an additional 6 hours. After treatments, the wells were washed, stained, and quantified using the same crystal violet staining protocol as described above.

### Assessment of phage-mediated growth inhibition of *V. parahaemolyticus* 108T

To evaluate the inhibitory effects of phages on bacterial growth, log-phase cultures of wild-type *V. parahaemolyticus* 108T were mixed with filtered phage lysates (single and combined) at MOIs of 0.01, 0.1, 1, and 10. The phage-bacteria mixtures (800 µL) were dispensed into 48-well microplates, with phage-free bacterial cultures serving as controls. Bacterial growth kinetics were monitored by measuring an optical density at 600 nm (OD_600_) every 30 minutes for 12 hours using a TECAN Infinite series microplate reader (74).

### Phage DNA extraction, sequencing, and bioinformatics analysis

The phage DNA was extracted according to the phenol-chloroform method described previously (89, 90). One milliliter of purified phage suspension was treated with 1.5 µL of proteinase K (100 mg/mL), 100 µL of SDS (10% wt/vol), and 10 µL of EDTA (0.5 M, pH 8.0); after mixing, the reactions were incubated at 55°C for 3 hours to disrupt the phage capsids. This was followed by DNA extraction with phenol:chloroform:isoamyl alcohol solution (25:24:1, vol/vol) and chloroform:isoamyl alcohol solution (24:1, vol/vol). The DNA was precipitated with 50% isopropanol (vol/vol) and centrifuged at 12,000 × *g* for 15 minutes at 4°C. The DNA pellet was then washed twice using 1 mL of ice-cold 70% ethanol, air-dried, and resuspended in 100 µL of 1× TE buffer and stored at −20°C. The high-throughput sequencing of viral genomes was then performed using shotgun method on the Illumina HiSeq 2500 platform from Oebiotech Co. (Qingdao, China), and the quality-filtered paired-end reads, which were quality-controlled and removed from host contamination, were assembled using SPAdes 3.14.1 (91). The GeneMarkS online server (https://exon.gatech.edu/GeneMark/genemarks.cgi) (92) was used to predict ORFs in the assembled phage sequences, after which translated ORFs were functionally annotated through homology search by BLASTp (https://www.ncbi.nlm.nih.gov/) with default settings and a threshold *E* value of  <1*e*^−5^. Meanwhile, the online HHpred server (https://toolkit.tuebingen.mpg.de/tools/hhpred) (93) against the PDB_mmCIF70_8_Mar, Pfam-A_v36, UniProt-SwissProt-viral70_3_Nov_2021, and NCBI_Conserved_Domains (CD)_v3.19 was employed through homology detection and structure prediction to support the annotation process. Additionally, tRNA genes were predicted by tRNAscan-SE Search Server (https://lowelab.ucsc.edu/tRNAscan-SE/) (94). The potential antibiotic genes and virulence factors were identified using the online databases Comprehensive Antibiotic Resistance Database (https://card.mcmaster.ca/analyze) (95) and Virulence Factor Database http://www.mgc.ac.cn/VFs/search_VFs.htm) (96), respectively. Genomes were visualized with the Proksee server (https://proksee.ca/) (97). The amino acid sequences of phages VpT and VpR were uploaded to the ViPTree 4.0 server (https://www.genome.jp/viptree) (98) for proteomic tree construction. ANI was calculated using the VIRIDIC web service (https://rhea.icbm.uni-oldenburg.de/viridic/) (99) to compute pairwise intergenomic similarities among viral genomes.

### Statistical analysis

All quantitative data are presented as the mean ± the standard deviations from three independent biological replicates (*n* = 3). Statistical significance was measured using one-way analysis of variance with Tukey’s multiple comparisons test using GraphPad Prism 10.1.2. *P* < 0.05 was considered significant.

## Data Availability

The complete genome sequences of phages VpT and VpR were deposited at NCBI GenBank under accession numbers PV847829 and PV847830, respectively.

## SUPPLEMENTAL MATERIAL

Supplemental material aem.01673-25-s0001.docx Supplemental methods, Fig. S1 to S3, and Tables S1 to S4.

ASM does not own the copyrights to Supplemental Material that may be linked to, or accessed through, an article. The authors have granted ASM a non-exclusive, world-wide license to publish the Supplemental Material files. Please contact the corresponding author directly for reuse.
