# Supplementary material for: Enhanced control of pathogenic Vibrio spp. in aquaculture using phages capable of disrupting biofilms outside their host range
Source: Appl Environ Microbiol. 2025 Oct 22;91(11):e01673-25. doi: 10.1128/aem.01673-25 (PMC12628691; doi:10.1128/aem.01673-25)
Supplement: Supplemental material — Supplemental methods, Fig. S1 to S3, and Tables S1 to S4. [file aem.01673-25-s0001.docx]

**Supporting Information for**

**Enhanced control of pathogenic *Vibrio* spp. in aquaculture using phages capable of disrupting biofilms outside their host range**

Ni Wang^1,2^†, Chengcheng Li^2,4^†^*^, Jiulong Zhao^2,4^, Yufei Yue^2,4^, Tongmei Shi^2,4^, Zengmeng Wang^2,4^, Yantao Liang^1^, Yongyu Zhang^2,3,4*^, Min Wang^1,5*^

^1^College of Marine Life Sciences, Institute of Evolution and Marine Biodiversity, MoE Laboratory of Evolution and Marine Biodiversity, Frontiers Science Center for Deep Ocean Multispheres and Earth System, Center for Ocean Carbon Neutrality, Ocean University of China, Qingdao, China

^2^Qingdao New Energy Shandong Laboratory, Key Laboratory of Biofuels, Shandong Provincial Key Laboratory of Energy Genetics, Qingdao Institute of Bioenergy and Bioprocess Technology, Chinese Academy of Sciences, Qingdao 266101, China

^3^Southern Marine Science and Engineering Guangdong Laboratory (Zhuhai), Zhuhai 519000, China

^4^University of Chinese Academy of Sciences, Beijing 100049, China

^5^Haide College, Ocean University of China, Qingdao, China

***Correspondence**:

Chengcheng Li, licc@qibebt.ac.cn; Yongyu Zhang, zhangyy@qibebt.ac.cn; Min Wang, mingwang@ouc.edu.cn

† Ni Wang and Chengcheng Li contributed equally to this work.

**This file includes:**

Supplementary Methods

Figures S1-S3

Tables S1-S4

**Supplementary Methods**

***Phage adsorption kinetics***

Phage adsorption efficiency was assessed using a modified protocol based on a previously described method (1). Briefly, log-phase cultures of wild-type *Vibrio parahaemolyticus* strain 108T and its phage-resistant derivative 108R (OD_600_ = 0.6) were individually incubated with phages at an MOI of 0.01 in RO medium (28°C, 160 rpm). At 20-minute intervals, 1 mL aliquots were collected, filtered through 0.22-μm sterile membranes and serially diluted. The titers of unadsorbed phage were quantified by spot assay.

**Supplemental References**

1. **Naknaen A, Samernate T, Wannasrichan W, Surachat K, Nonejuie P, Chaikeeratisak V**. 2023. Combination of genetically diverse *Pseudomonas* phages enhances the cocktail efficiency against bacteria. *Sci Rep* 13:8921.

**Supplementary Figures**


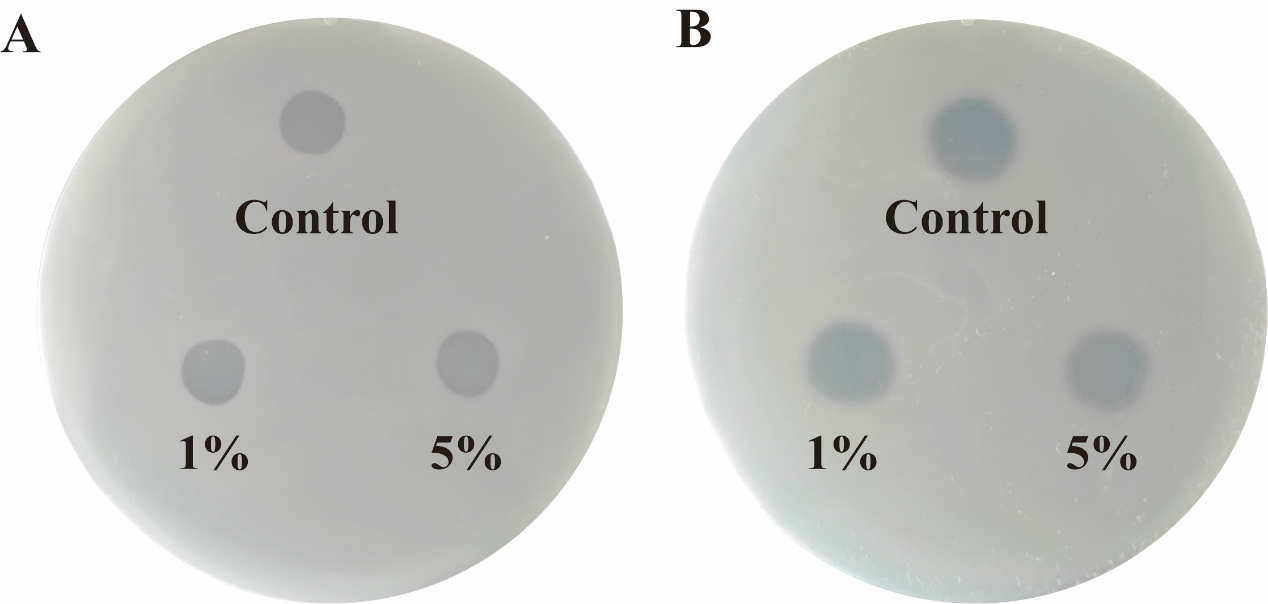


**Fig. S1** **Chloroform sensitivity of phage VpT (A) and phage VpR (B).**


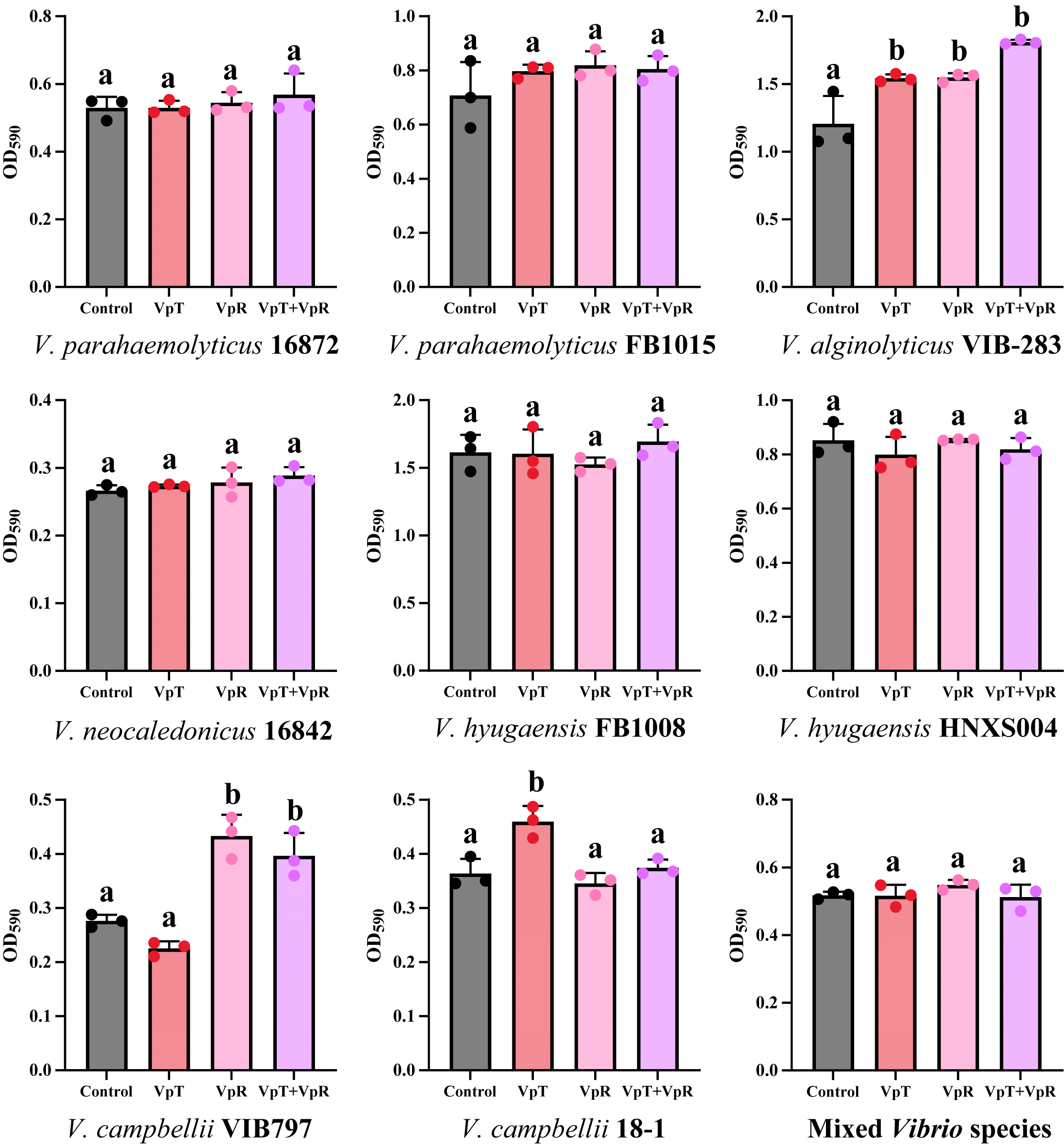


**Fig. S2 Preventive effects of phages VpT, VpR, and VpT/VpR cocktail (MOI = 10) on biofilm formation by various non-host *Vibrio* species and mixed-species *Vibrio* communities.** Different letters represent significant statistical differences (*p* < 0.05) between groups.


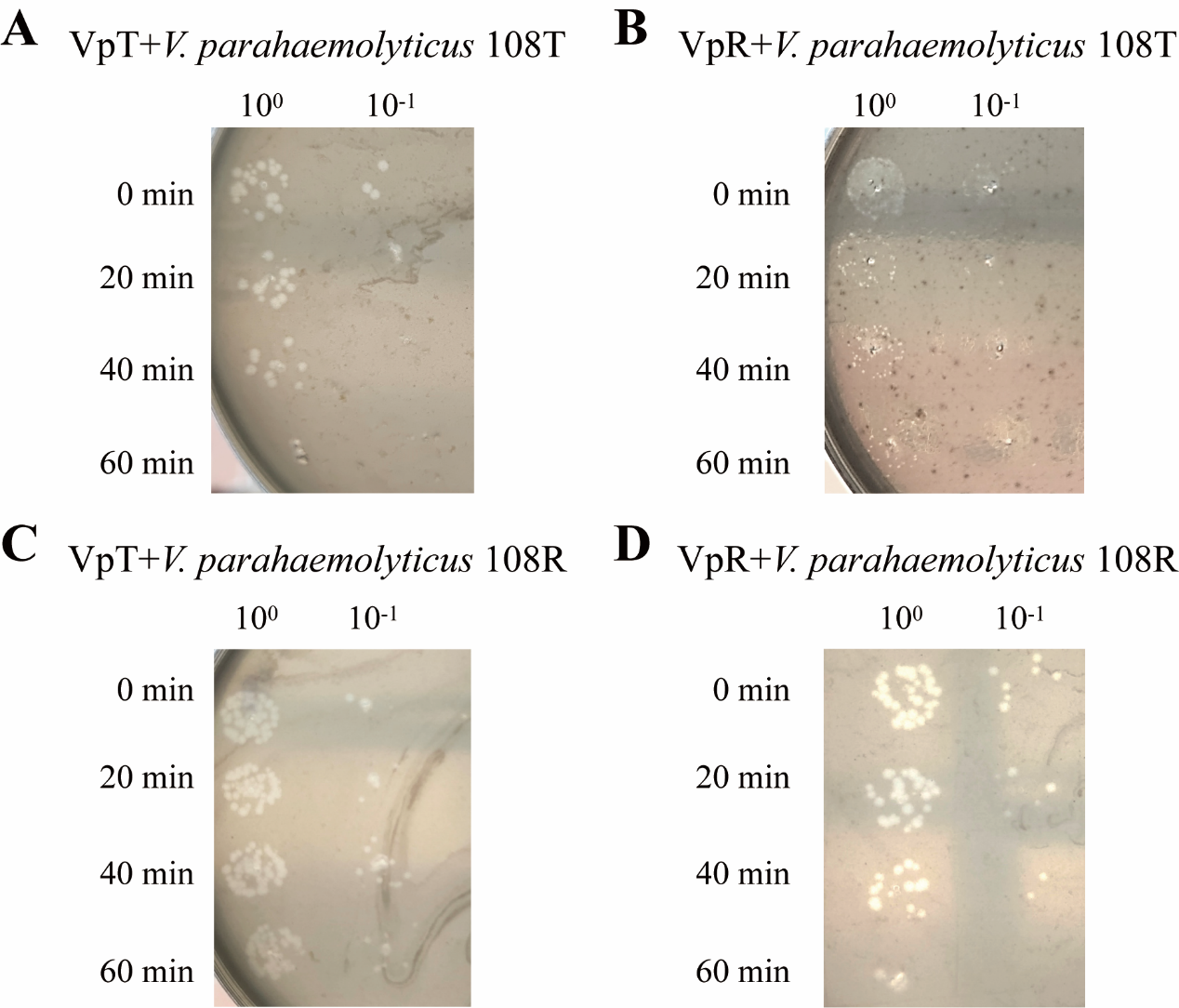


**Fig. S3** **Spot assay of phage adsorption kinetics.** The decline in extracellular phage titers over time (e.g., VpT + *V. parahaemolyticus* 108T, VpR + *V. parahaemolyticus* 108T, and VpR + *V. parahaemolyticus* 108R) indicates successful adsorption of phages to their respective hosts. In contrast, the stable phage titers observed in the VpT + *V. parahaemolyticus* 108R group suggest a failure of phage adsorption.

**Supplementary Tables**

**Table S1** **Antibiotic susceptibility profile of *V. parahaemolyticus* 108T.**

| Antimicrobials | Content of dispersion (μg/pill) | Results |
| --- | --- | --- |
| Ceftriaxone | 30 | S |
| Oxacillin | 1 | R |
| Cefoperazone | 75 | I |
| Norfloxacin | 10 | I |
| Ampicillin | 10 | R |
| Amikacin | 30 | R |
| Ofloxacin | 5 | S |
| Carbenicillin | 100 | R |
| Gentamicin | 10 | S |
| Ciprofloxacin | 5 | I |
| Piperacillin | 100 | R |
| Kanamycin | 30 | I |
| Vancomycin | 30 | R |
| Cefazolin | 30 | S |
| Tetracycline | 30 | R |
| Compound sulfamethoxazole (SMZ/ TMP) | 23.75/1.25 | S |
| Doxycycline | 30 | I |
| Cefuroxime | 30 | I |
| Minocycline | 30 | R |
| Chloramphenicol | 30 | S |
| Ceftazidime | 30 | I |

Table S1 Cont)

| Antimicrobials | Content of dispersion (μg/pill) | Results |
| --- | --- | --- |
| Penicillin | 10 U/pill | R |
| Clindamycin | 2 | R |

Key: S, Susceptible; I, Intermediate; R, Resistant

**Table S2 Basic genome information of phages VpT and VpR.**

| Phage name | VpT | VpR |
| --- | --- | --- |
| Genome length (bp) | 48,359 | 45,755 |
| GC content (%) | 47.99 | 52.25 |
| No. of ORFs | 73 | 60 |
| Functionally annotated ORFs | 32 | 31 |
| Circular or linear | Circular | Circular |
| tRNA genes | 1 | 1 |
| Antibiotic resistance genes | No | No |
| Virulence genes | No | No |
| Morphotypes | podovirus | podovirus |
| GenBank accession numbers | PV847829 | PV847830 |

**Table S3 Functionally-annotated ORFs in the genome of phage VpT.**

| ORF | LeftEnd | RightEnd | Strand | Putative Function |
| --- | --- | --- | --- | --- |
| 1 | 2 | 1276 | - | helicase |
| 2 | 1282 | 1527 | - | DNA helicase/primase |
| 5 | 2287 | 2694 | - | gamma-glutamyl cyclotransferase |
| 6 | 2694 | 3497 | - | ATP-grasp enzyme |
| 8 | 3705 | 5393 | - | L-glutamine-D-fructose-6-phosphate aminotransferase |
| 9 | 5393 | 7180 | - | glutamine amidotransferase |
| 11 | 7766 | 8569 | - | COOH.NH2 ligase |
| 27 | 14897 | 15448 | - | coil containing protein |
| 32 | 18290 | 18778 | + | terminase small subunit |
| 33 | 18775 | 19107 | + | endolysin |
| 35 | 19606 | 21069 | + | terminase large subunit |
| 37 | 21425 | 23524 | + | portal protein |
| 39 | 23781 | 24842 | + | capsid and scaffold protein |
| 40 | 24858 | 25811 | + | major head protein |
| 42 | 26186 | 26839 | + | virion structural protein |
| 44 | 27836 | 28786 | + | tail fiber domain-containing protein |
| 47 | 29339 | 29983 | + | tail fibers protein |
| 48 | 29994 | 31544 | + | virion structural protein |
| 51 | 32258 | 33190 | + | tail structure protein |
| 54 | 34165 | 35349 | + | virion structural protein |
| 55 | 35365 | 38481 | + | putative structural protein |
| 57 | 38794 | 39315 | - | NUDIX hydrolase |
| 59 | 39611 | 40381 | - | constituent protein |

Table S3 Cont)

| ORF | LeftEnd | RightEnd | Strand | Putative Function |
| --- | --- | --- | --- | --- |
| 61 | 40574 | 40744 | - | endonuclease |
| 63 | 41836 | 42699 | - | exonuclease |
| 65 | 43195 | 43620 | - | endolysin |
| 66 | 43604 | 44170 | - | Gp2.5-like ssDNA binding protein and ssDNA annealing protein |
| 68 | 44432 | 46072 | - | DNA polymerase |
| 69 | 46053 | 46463 | - | 5'-nucleotidase |
| 70 | 46460 | 46762 | - | HNH endonuclease |
| 72 | 47327 | 47830 | - | DNA polymerase |
| 73 | 47865 | 48359 | - | helicase |

**Table S4 Functionally-annotated ORFs in the genome of phage VpR.**

| ORF | LeftEnd | RightEnd | Strand | Putative Function |
| --- | --- | --- | --- | --- |
| 1 | 1647 | 2105 | + | terminase small subunit |
| 2 | 2137 | 2538 | + | lysozyme |
| 3 | 2538 | 2873 | + | endolysin |
| 4 | 2875 | 4320 | + | terminase large subunit |
| 5 | 4321 | 6441 | + | portal protein |
| 7 | 6677 | 7681 | + | scaffolding protein |
| 8 | 7693 | 8637 | + | major head protein |
| 10 | 8995 | 9633 | + | virion structural protein |
| 12 | 10223 | 13330 | + | AAA family ATPase |
| 17 | 14247 | 14867 | + | tail fiber protein |
| 18 | 14871 | 16451 | + | structural protein |
| 19 | 16451 | 16735 | + | constituent protein |
| 21 | 17224 | 18171 | + | tail structure protein |
| 23 | 18556 | 20190 | + | structural protein |
| 24 | 20194 | 23325 | + | structural protein |
| 26 | 23606 | 24364 | - | metallophosphoesterase |
| 27 | 24351 | 24629 | - | endonuclease |
| 28 | 24598 | 25554 | - | endonuclease I |
| 29 | 25541 | 26482 | - | 5'-3' exonuclease |
| 32 | 27055 | 27462 | - | endolysin |
| 33 | 27475 | 28065 | - | DNA binding protein |
| 34 | 28137 | 29780 | - | DNA polymerase |
| 39 | 31032 | 31307 | - | DNA polymerase |
| 40 | 31304 | 31813 | - | DNA polymerase exonuclease subunit |

Table S4 Cont)

| ORF | LeftEnd | RightEnd | Strand | Putative Function |
| --- | --- | --- | --- | --- |
| 41 | 31797 | 33500 | - | primase/helicase |
| 43 | 33856 | 34287 | - | gamma-glutamyl cyclotransferase |
| 44 | 34287 | 35321 | - | ribosomal protein S6 glutaminyl transferase |
| 46 | 35568 | 37094 | - | glutamine amidotransferase |
| 47 | 37127 | 38320 | - | amidoligase |
| 49 | 38865 | 39653 | - | COOH.NH2 ligase |
| 53 | 41797 | 42627 | - | transposase |
